# Daptomycin Resistance Occurs Predominantly in *vanA*-Type Vancomycin-Resistant *Enterococcus faecium* in Australasia and Is Associated With Heterogeneous and Novel Mutations

**DOI:** 10.3389/fmicb.2021.749935

**Published:** 2021-10-20

**Authors:** Lucy Li, Charlie Higgs, Adrianna M. Turner, Yi Nong, Claire L. Gorrie, Norelle L. Sherry, Kristin H. Dyet, Torsten Seemann, Deborah A. Williamson, Timothy P. Stinear, Benjamin P. Howden, Glen P. Carter

**Affiliations:** ^1^Department of Microbiology and Immunology, Peter Doherty Institute for Infection and Immunity, The University of Melbourne, Melbourne, VIC, Australia; ^2^Antimicrobial Reference and Research Unit, Microbiological Diagnostic Unit Public Health Laboratory, Peter Doherty Institute for Infection and Immunity, The University of Melbourne, Melbourne, VIC, Australia; ^3^Department of Infectious Diseases, Austin Health, Melbourne, VIC, Australia; ^4^The Institute of Environmental Science and Research, Porirua, New Zealand

**Keywords:** *Enterococcus faecium*, daptomycin-resistance, *vanA*, bacteraemia, single nucleotide polymorphisms (SNPs)

## Abstract

Healthcare associated infections caused by vancomycin-resistant *Enterococcus faecium* (VREfm) have a major impact on health outcomes. VREfm is difficult to treat because of intrinsic and acquired resistance to many clinically used antimicrobials, with daptomycin being one of the few last line therapeutic options for treating multidrug-resistant VREfm. The emergence of daptomycin-resistant VREfm is therefore of serious clinical concern. Despite this, the impact that daptomycin-resistant VREfm have on patient health outcomes is not clearly defined and knowledge on the mechanisms and genetic signatures linked with daptomycin resistance in VREfm remains incomplete. To address these knowledge gaps, phenotypic daptomycin susceptibility testing was undertaken on 324 *E. faecium* isolates from Australia and New Zealand. Approximately 15% of study isolates were phenotypically resistant to daptomycin. Whole genome sequencing revealed a strong association between *vanA*-VREfm and daptomycin resistance, with 95% of daptomycin-resistant study isolates harbouring *vanA*. Genomic analyses showed that daptomycin-resistant VREfm isolates were polyclonal and carried several previously characterised mutations in the *liaR* and *liaS* genes as well as several novel mutations within the *rpoB, rpoC*, and *dltC* genes. Overall, 70% of daptomycin-resistant study isolates were found to carry mutations within the *liaR, rpoB, rpoC*, or *dltC* genes. Finally, in a mouse model of VREfm bacteraemia, infection with the locally dominant daptomycin-resistant clone led to reduced daptomycin treatment efficacy in comparison to daptomycin-susceptible *E. faecium*. These findings have important implications for ongoing VREfm surveillance activities and the treatment of VREfm infections.

## Introduction

*Enterococcus faecium*, in particular vancomycin-resistant *E. faecium* (VREfm) are important nosocomial pathogens. VREfm can be very difficult to treat, are easily transmitted within the hospital environment and have a high propensity for horizontal gene transfer and recombination, which has contributed to the emergence of multidrug-resistant (MDR) isolates (Carter et al., 2016). VREfm is a World Health Organization (WHO) priority pathogen (World Health Organization [WHO], 2017).

Daptomycin is a lipopeptide antibiotic with potent bactericidal activity against *E. faecium* and is considered a last line treatment for VREfm (Beriashvili et al., 2018), along with linezolid and tigecycline (Gorrie et al., 2019). Recent reports documenting the emergence of daptomycin-resistant *E. faecium* are therefore of serious concern (Kamboj et al., 2011; Munita et al., 2012; Douglas et al., 2019). Despite the importance of daptomycin as a therapeutic option for MDR isolates, the mechanisms associated with resistance in *E. faecium* are not completely defined, and the genetic signatures and clinical consequences associated with daptomycin-resistant *E. faecium* are not well understood.

The best studied mechanism of daptomycin resistance in VREfm involves the LiaFSR three component regulatory system, with single nucleotide polymorphisms (SNPs) within the *liaFSR* genes identified in many daptomycin-resistant clinical isolates (Munita et al., 2012). However, several studies have noted that *liaFSR* mutations do not always result in daptomycin resistance and many daptomycin-resistant isolates contain wild-type *liaFSR* genes (Werth et al., 2014; Lellek et al., 2015), indicating that other molecular pathways might also be important.

Here we report a strong association between *vanA*-type VREfm and daptomycin resistance, and the identification of several novel mutations potentially involved in the development of daptomycin resistance in *E. faecium*. Finally, in a murine model of VREfm bacteraemia, we show that daptomycin treatment efficacy is compromised in animals infected with daptomycin-resistant VREfm in comparison to daptomycin-susceptible VREfm.

## Materials and Methods

### Bacterial Strains and Growth Conditions

A total of 324 clinical *E. faecium* isolates (Supplementary Table 1) from the Microbiological Diagnostic Unit Public Health Laboratory (MDU PHL) culture collection were randomly selected from a list of anonymised isolate numbers with only the country of origin known. The isolates were part of a larger *E. faecium* collection of approximately 2000 isolates amassed by MDU PHL as part of ongoing Victorian public health surveillance activities and research activities that include isolates sent from Victoria, New Zealand, and New South Wales diagnostic laboratories. Isolates within the MDU PHL collection are both clinical and screening isolates. Individual patient information relating to the study isolates was not accessible. All study isolates were collected between 2007 and 2018, with 174 isolates originating in Australia and 150 isolates being from New Zealand. Study isolates were deemed to be representative of the broader MDU PHL VREfm collection in terms of collection year, van-status and ST types.

Unless otherwise stated, isolates were maintained on horse blood agar and grown in brain heart infusion (BHI) broth at 37°C.

Daptomycin (Cubicin) was purchased from Merck, Sharpe, and Dohme.

### Daptomycin Broth Microdilution Assay

Daptomycin susceptibility testing was performed using broth microdilution minimum inhibitory concentration assays according to CLSI methods [Clinical and Laboratory Standards Institute (CLSI), 2015]. In a 96-well plate, a two-fold dilution series (from 32 to 0.5 mg/L) of daptomycin was made in 100 μL volumes of cation adjusted Mueller-Hinton w/TES broth (CAMHBT) (Thermo Fisher) additionally supplemented with 50 mg/L Ca^2+^. An inoculum of 100 μL overnight *E. faecium* broth culture adjusted to 1 × 10^6^ cfu/mL in CAMHBT supplemented with 50 mg/L Ca^2+^ was then added to each well. After 24 h incubation, the MIC was defined as the lowest antimicrobial concentration that inhibited visible growth. All assays were performed in biological triplicate, with the median MIC value reported. In accordance with recent guidelines (Humphries, 2019) isolates with a daptomycin MIC of ≥8 mg/L were considered to be daptomycin-resistant. If there was a major MIC discrepancy within triplicate assays for an isolate whereby one or two repeats gave a susceptible MIC (≤4 mg/L) and the remaining repeat gave a resistant MIC (≥8 mg/L) (or vice versa), then an additional four biological replicates were performed with the median value of 7 independent repeats then reported. Note that a major discrepancy of this sort was only observed with five isolates, all of which were subsequently reported as susceptible after additional replicates were performed.

### Whole Genome Sequencing

Bacterial genomic DNA was extracted using a JANUS automated workstation (PerkinElmer) and Chemagic magnetic bead technology (PerkinElmer). Genomic DNA libraries were prepared using the Nextera XT kit (Illumina Inc.). Whole genome sequencing (WGS) was performed on the Illumina NextSeq platform using 2 × 150 bp paired end chemistry as before (Carter et al., 2016).

The genomic dataset generated for this study can be found in the ENA repository associated with bioprojects PRJNA433676, PRJNA565795, PRJEB23767, PRJNA565795, and PRJEB47276.

### Bioinformatic Analyses

The genomes of daptomycin-resistant study isolates (Supplementary Table 1) underwent *de novo* assembly using Shovill (v.1.0.1),^1^ and genome annotation was achieved using Prokka (v. 1.13.3) (Seemann, 2014). Short read sequence data from daptomycin-resistant isolates were mapped against the reference DO genome (TX16) (Diaz et al., 2014) and SNPs identified with Snippy (v. 4.6.0).^2^ The resulting alignment comprising 16,577 core SNP sites (core alignment length: 1,857,904), was used to infer the phylogeny using the maximum likelihood method with the general time reversible model using constant sites [IQtree (v.2.1.2) (Nguyen et al., 2015)]. Recombination masking [gubbins (v.2.4.1) (Croucher et al., 2014)] was performed however the resulting alignment was only made up of 983 core SNP sites and so was deemed too small to be used to build the phylogeny. A core SNP alignment, comprising 22,081 SNP sites (core alignment length: 1,373,512), was also generated for Australian study isolates and was used to build a phylogeny as above. Two outlier isolates (DMG1901764 and DMG1901754) were removed from the core alignment due to their abnormally large distance from the reference. This alignment was not masked for recombination. Phylogenetic trees were visualised using ggtree (Yu et al., 2017) in R studio (v4.0.2).^3^

*In silico* multi-locus sequence types (MLST) were determined using the mlst tool (v. 2.10)^4^ and the *E. faecium* pubMLST database).^5^

The *vanA* and *vanB* vancomycin resistance genes, were identified *in silico* using ABRicate (v. 1.0.1)^6^ and the Resfinder database (Zankari et al., 2012) using the default settings.

### Murine Vancomycin-Resistant *Enterococcus faecium* Bacteraemia Model

Animal experimentation was carried out with approval from the University of Melbourne, Department of Biochemistry and Molecular Biology, Dental Science, Medicine, Microbiology & Immunology, and Surgery Animal Ethics Committee. A total of 6–8 weeks old female C57BL/6 mice were infected with either the daptomycin-susceptible strain DMG1800332, or daptomycin-resistant clinical isolates DMG1901766, DMG1700661 (Carter et al., 2018), or DMG1700787. Mice were infected via intraperitoneal injection with 10^9^ CFU resuspended in sterile rat faecal extract (SRFE) (Singh et al., 1998). Controls were mock infected with SRFE only. Each strain was used to infect two groups of animals. Two hours post-infection, one group of animals was administered daptomycin (50 mg/kg) by subcutaneous injection [this results in similar exposure (AUC_0__–__24_) to that observed in humans receiving 8 mg/kg of daptomycin] (Heine et al., 2010) and the other group received saline (vehicle) by subcutaneous injection. At 24 h post infection, mice were killed by CO_2_ asphyxiation and blood collected by cardiac puncture into EDTA tubes before being washed and serially diluted in PBS. Dilutions were then plated onto ChromeID VRE agar (BioMerieux) for enumeration of VREfm in the blood.

Statistical significance was calculated using a non-parametric Mann–Whitney U-test using GraphPad Prism 9.0.1.

## Results

### Daptomycin Resistance Is Common in Clinical Australian Vancomycin-Resistant *Enterococcus faecium* Isolates

**FIGURE 1:**
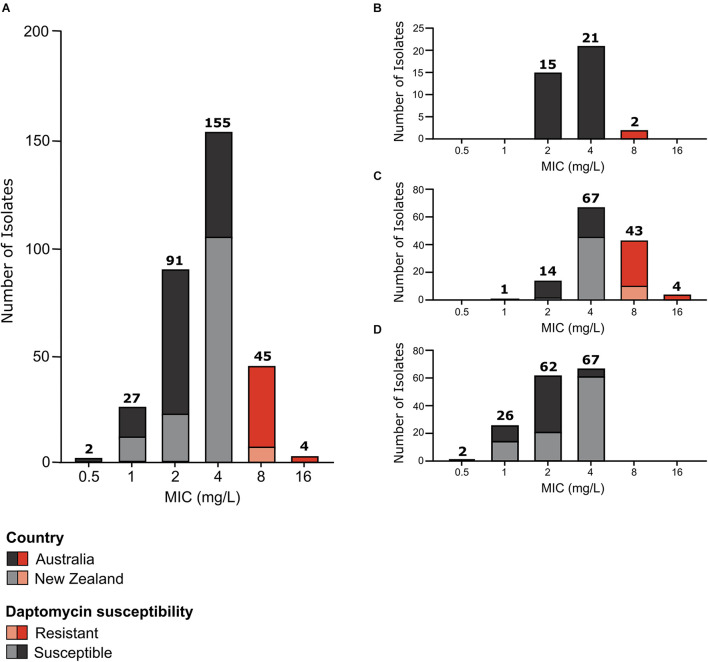
Daptomycin susceptibility of *E. faecium* study isolates. Daptomycin MIC distribution of **(A)** all study isolates (*n* = 324); **(B)** VSEfm isolates (*n* = 38); **(C)** *vanA*-VREfm isolates (*n* = 129); and **(D)** *vanB*-VREfm isolates (*n* = 157). Australian isolates are represented by dark grey and dark red blocks and New Zealand isolates are represented by light grey and light red blocks. Daptomycin-susceptible MICs are shown in grey (MIC < 8 mg/L) and daptomycin-resistant MICs are highlighted in red (MIC ≥ 8 mg/L). The number of isolates with each MIC value shown on the *x*-axis is displayed above the respective bars.

A random selection of 324 isolates was made from a larger unbiased collection of Australian and New Zealand isolates stored at the MDU PHL. These isolates comprised *vanB*-VREfm (*n* = 157), *vanA-*VREfm (*n* = 129), and vancomycin-susceptible *E. faecium* (VSEfm) (*n* = 38). All isolates underwent phenotypic daptomycin-susceptibility testing (Figures 1A–D).

Overall, the isolates displayed a unimodal wild-type MIC distribution (Figure 1A) with a mode MIC of 4 mg/L. When analysed by country, a unimodal MIC distribution was evident for both Australian and New Zealand isolates, with a mode MIC of 2 mg/L for Australian isolates and 4 mg/L for New Zealand isolates (Figure 1A). The MIC_50_ and MIC_90_ for Australian isolates was 4 and 8 mg/L, respectively, while New Zealand isolates had an MIC_50_ and MIC_90_ of 4 mg/L.

In total 49 daptomycin-resistant *E. faecium* isolates were identified according to current guidelines (Humphries, 2019) (MIC of ≥8 mg/L). This represents a resistance rate of 15.1% for the isolates tested. Although similar numbers of Australian and New Zealand *E. faecium* were tested (175 isolates from Australia and 150 isolates from New Zealand), the majority of daptomycin-resistant isolates were from Australia, with 39 isolates being from Australia and just 10 isolates from New Zealand.

### Daptomycin Resistance Is Strongly Associated With *vanA*-Type Vancomycin-Resistant *Enterococcus faecium*

Strikingly, when analysed by *E. faecium* strain type (i.e. *vanA*-VREfm, *vanB*-VREfm, or VSEfm) a strong association between *vanA*-VREfm and daptomycin resistance was observed (Figure 1), with 96% of daptomycin-resistant isolates being of the *vanA*-VREfm type and 4% being VSEfm. No daptomycin-resistant *vanB*-VREfm study isolates were identified. Statistical analysis showed that *vanA*-VREfm were significantly more daptomycin-resistant than *vanB*-VREfm (*p* < 0.0001; Fisher’s exact test) and VSEfm (*p* < 0.0001; Fisher’s exact test), with MIC_50_ values of 4, 2, and 4 mg/L for *vanA*-VREfm, *vanB*-VREfm, and VSEfm, respectively, and MIC_90_ values of 8, 4, and 4 mg/L, respectively. Country of origin did not influence the correlation between daptomycin resistance and *vanA*-VREfm (Figure 1).

### Daptomycin Resistance Occurs in Phylogenetically Diverse *Enterococcus faecium* Clinical Isolates

**FIGURE 2:**
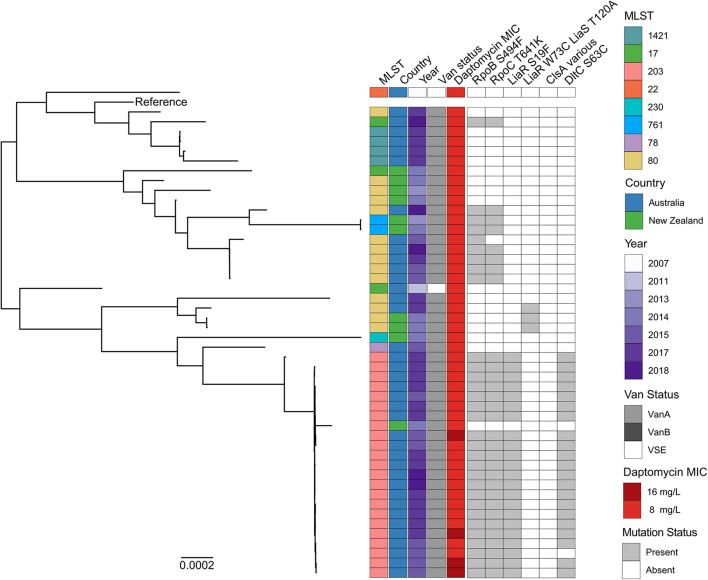
Maximum likelihood core-SNP phylogeny of 49 Australian and New Zealand daptomycin-resistant *E. faecium* isolates. Coloured blocks show the multi-locus sequence type (MLST), country of origin (country), year of isolation (year), van status (i.e., *vanA*, *vanB*, and VSE) and daptomycin MIC (8 and 16 mg/L) for each isolate within the phylogeny. Grey blocks show the presence of the RNAP β-subunit S494F mutation (RpoB S494F), the RNAP β′-subunit T641K mutation (RpoC T641K), the LiaR S19F mutation, the DltC S63C mutation, the LiaR W73C and LiaS T120A co-occurring mutations or mutations within ClsA previously associated with daptomycin resistance in VREfm (ClsA various). The *vanA*-VREfm strain D0 (TX16) (Diaz et al., 2014) was used as the reference.

Given the strong association of *vanA*-VREfm and daptomycin-resistance it was important to determine whether the isolates were clonal. MLST analysis showed that daptomycin-resistance occurred in eight different sequence types (STs); ST203, ST80, ST1421, ST17, ST761, ST78, ST230, and ST22 (Figure 2). Our analyses showed that each ST formed a monophyletic clade, with the exception of ST80 and ST17, which were paraphyletic. ST203 was the predominant type, comprising 47% (*n* = 23/49) of daptomycin-resistant isolates. ST80 also accounted for a large proportion (29%, *n* = 14/49) of resistant isolates. The remaining daptomycin-resistant isolates consisted of four ST1421 isolates (8%), three ST17 isolates (6%), two ST761 isolates (4%), and a single isolate (2%) for each of ST78, ST230, and ST22.

To understand the phylogenetic relatedness of daptomycin-resistant study isolates, a maximum likelihood phylogeny was constructed (Figure 2). Substantial genetic diversity was evident, with a mean core genome alignment pairwise SNP distance of 4,161 SNPs (range 0–9,231 SNPs). The population structure was polyphyletic, with multiple distinct clades evident, generally summarised by previously assigned multi-locus ST.

Despite this overall level of diversity among resistant isolates, isolates from the predominant ST203 group displayed more restricted diversity, and had a mean pairwise SNP distance of 94 core genome SNPs (range 0–872 SNPs). There was evidence of clonal spread within the Australian ST203 clade, with 21 out of 23 isolates differing by no more than 39 core genome SNPs. In contrast, isolates within the second largest daptomycin-resistant group (ST80) were more genetically diverse, with a mean pairwise SNP distance of 3,600 SNPs (range 0–7178 SNPs). Notably, daptomycin-resistant ST80 isolates clustered throughout the tree within four distinct sub-clades, some containing both Australian and New Zealand isolates.

These data collectively suggest that daptomycin resistance in Australian and New Zealand VREfm clinical isolates has arisen independently and is not the consequence of a recent clonal outbreak.

### Novel Mutations Are Associated With Daptomycin Resistance in Australian and New Zealand Clinical Vancomycin-Resistant *Enterococcus faecium* Isolates

To understand the genetic changes associated with daptomycin resistance in the Australian and New Zealand study isolates, whole genome sequences from each resistant strain were analysed to identify any known *E. faecium* daptomycin resistance mutations. This analysis led to the identification of three isolates (6%) carrying the well-characterised W73C and T120A mutations in the LiaR and LiaS proteins respectively, and 22 isolates (44%) that harbour the less common S19F mutation in LiaR (Sorlózano et al., 2015). The isolates carrying the T120A and W73C LiaSR mutations were all ST80, with 2 of the isolates being from New Zealand and 1 from Australia (Figure 2). All 22 isolates carrying the S19F mutation in LiaR clustered within the ST203 clade and were of Australian origin (Figure 2). No other mutations previously linked with daptomycin resistance in *E. faecium* (shown in Supplementary Table 2) were identified, including within the *clsA* gene (Humphries et al., 2012; Tran et al., 2013; Lellek et al., 2015; Wang et al., 2018; Prater et al., 2019). Three novel SNPs (relative to the reference strain *E. faecium* D0) in genes associated with daptomycin resistance in *Staphylococcus aureus* were however identified. These mutations were S63C in DltC, which plays a role in D-alanylation of teichoic acid, a T641K mutation in the RNA polymerase (RNAP) β′-subunit, encoded by *rpoC* and a S494F mutation in the RNAP β-subunit, encoded by *rpoB*. These mutations were present in 22 (45%), 30 (61%), and 31 (63%) daptomycin-resistant isolates, respectively.

The DltC and LiaR mutations were found exclusively in Australian ST203 isolates, where they co-occurred with each other as well as the S494F and T641K mutations in RNAP (Figure 2). The RNAP mutations were more widespread and were present in both Australian and New Zealand isolates and from four different STs (ST203, ST80, ST17, and ST761). Notably, the RNAP mutations co-occurred in all but one isolate (AUSMDU00004090) irrespective of lineage.

**FIGURE 3:**
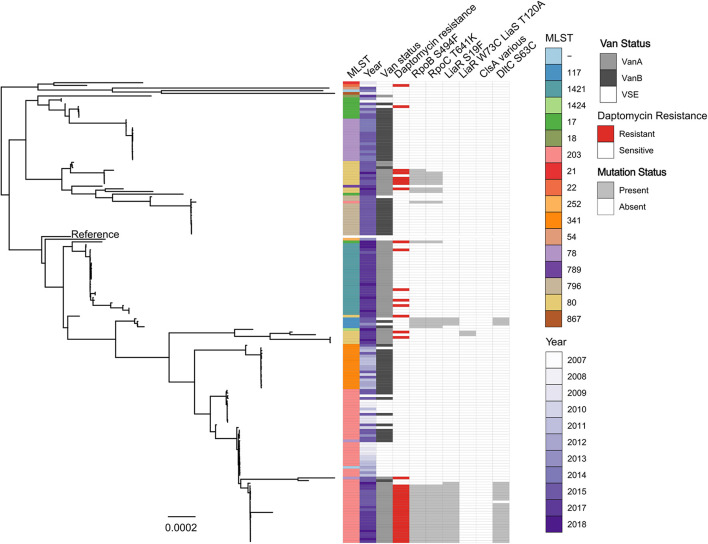
Maximum likelihood core-SNP phylogeny of 173 Australian *E. faecium* study isolates. Coloured blocks show the multi-locus sequence type (MLST), country of origin (country), year of isolation (year), *van* status (i.e., *vanA*, *vanB*, and VSE) and daptomycin status (resistant or susceptible) for each isolate within the phylogeny. Grey blocks show the presence of the RNAP β-subunit S494F mutation (RpoB S494F), the RNAP β′-subunit T641K mutation (RpoC T641K), the LiaR S19F mutation, the DltC S63C mutation, the LiaR W73C and LiaS T120A co-occurring mutations or mutations within ClsA previously associated with daptomycin resistance in VREfm (ClsA various) for each isolate within the phylogeny. The *vanA*-VREfm strain D0 (TX16) (Diaz et al., 2014) was used as the reference.

Given the high proportion of daptomycin-resistance among Australian VREfm isolates, a phylogenetic reconstruction of just Australian VREfm study isolates was made (Figure 3). Two daptomycin-susceptible isolates (DMG1901764 and DMG1901754) were excluded from this analysis as outliers (SNP distance from reference 58,442 and 53,355, respectively). This phylogenetic reconstruction showed that daptomycin-resistant isolates were distributed throughout the phylogeny and were generally interspersed with daptomycin-susceptible isolates of the same ST, providing further support for the hypothesis that daptomycin resistance may have emerged independently on multiple occasions within Australia. Daptomycin-resistant ST203 isolates were an exception to this general observation, with daptomycin-resistant ST203 isolates appearing to form a monophyletic clade that was separated for daptomycin-susceptible ST203 study isolates. With this in mind, it is perhaps noteworthy that all daptomycin-resistant ST203 study isolates carried *vanA*, while all susceptible isolates, with one exception (AUSMDU00012789), were either VSEfm or carried *vanB.*

The genomes of each Australian isolate (both daptomycin-resistant and -susceptible) were then interrogated for the presence of the RNAP β-subunit S494F, RNAP β′-subunit T641K, DltC S63C, and LiaR S19F mutations. There was a clear correlation between the presence of these mutations and daptomycin-resistance, as shown in Figure 3, with comparatively few daptomycin-susceptible isolates identified that carried these mutations. Overall, the S494F and T641K mutations in RNAP β- and β′-subunits respectively were found in 75% (29 out of 39 isolates) and 71% (28 out of 39 isolates) of daptomycin-resistant isolates respectively, compared to just 4.5% of daptomycin-susceptible isolates (6 out of 136 isolates). Similarly, the S19F mutation in LiaR and S63C mutations in DltC were identified in a high proportion (56 and 53%, respectively) of daptomycin-resistant isolates, but both mutations were much less abundant (3%; 4 of 136 isolates) in daptomycin-susceptible isolates. In comparison, the LiaR W73C and LiaS T120A mutations were identified in just 2.5% of daptomycin-resistant isolates (1 out of 39 isolates) and 0.73% of daptomycin-susceptible VREfm isolates (1 out of 136 isolates). Overall, approximately 77% (30 of 39 isolates) of Australian daptomycin-resistant VREfm study isolates carried mutations within the *liaR, rpoB, rpoC*, or *dltC* genes compared to 6% (8 or 136 isolates) of daptomycin-susceptible isolates. Spearman’s correlation test confirmed a significant correlation between daptomycin-resistant VREfm and the presence of the S494F RNAP β-subunit mutation (*p* < 0.0001), the T641K RNAP β′-subunit mutation (*p* < 0.0001), the S19F LiaR mutation (*p* < 0.0001) and the S63F DltC mutation (*p* < 0.0001). Given the low number of isolates carrying the LiaR W73C and LiaS T120A co-mutations it was not possible to determine whether they were significantly correlated with daptomycin-resistance or not.

Collectively these data suggest that specific mutations in LiaR, DltC, and RNAP, which are not commonly seen overseas, might play an important role in daptomycin resistance in Australian VREfm.

### Infection With the Dominant Australian Daptomycin-Resistant Vancomycin-Resistant *Enterococcus faecium* Clone Is Associated With Reduced Daptomycin Treatment Efficacy in a Murine Bacteraemia Model

The clinical impact of daptomycin-resistant VREfm on daptomycin treatment efficacy is currently not well defined. A murine model of VREfm bacteraemia was therefore used to determine whether infection with the predominant daptomycin-resistant clone identified in this study (ST203) would lead to reduced daptomycin treatment efficacy. Three ST203 daptomycin-resistant isolates (DMG1901766, DMG1700787, and DMG1700661), as well as a daptomycin-susceptible control strain (DMG1800332) were used to infect separate groups of mice. The daptomycin-susceptible control strain had a daptomycin MIC of 4 mg/L, while DMG1901766 had an MIC of 8 mg/L and DMG1700787 and DMG1700661 both had daptomycin MICs of 16 mg/L.

**FIGURE 4:**
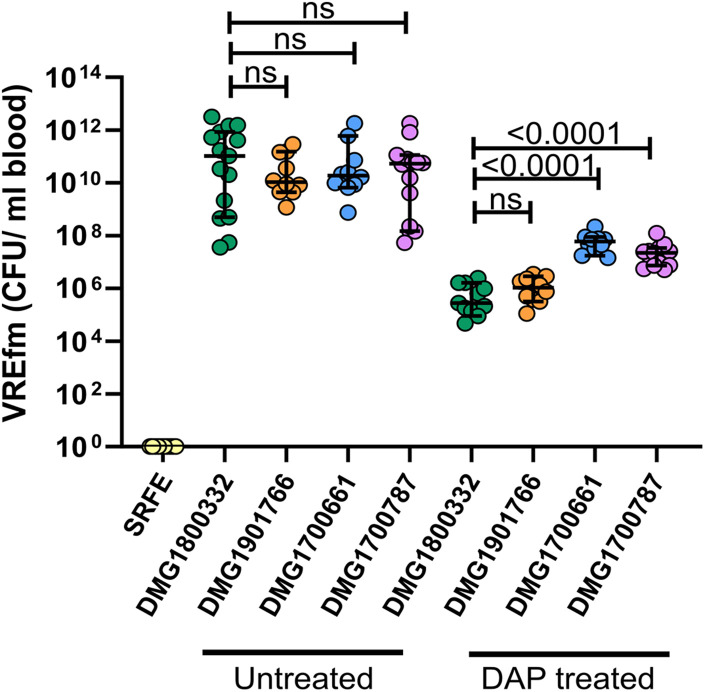
Efficacy of daptomycin treatment in a mouse model of VREfm bacteraemia. VREfm blood counts in uninfected mice administered sterile rate faecal extract (SRFE) or in mice infected with the daptomycin-susceptible isolate DMG1800332 (green) or daptomycin-resistant isolates DMG1901766 (orange), DMG1700661 (blue), or DMG1700787 (purple), and treated with either saline (untreated) or 50 mg/kg of daptomycin (DAP treated). Each coloured circle represents one animal (*n* = 10–15 mice), with the median value displayed by the horizontal black line. Error bars represent the 95% CI. *p*-Values were calculated using a Mann–Whitney non-parametric test and are shown for the indicated groups; ns indicates no significant difference between groups (*p* ≥ 0.05).

There was no significant difference in the level of each isolate in the blood of saline treated mice, with infected animals displaying a profound bacteraemia (bacterial counts ranging from 10^7^ to 10^12^CFU/mL of blood) regardless of the infecting isolate (Figure 4). As expected, a reduced level of VREfm was observed in the blood of animals treated with daptomycin in comparison to saline treated mice. Notably, under the conditions tested, there was more VREfm in the blood of daptomycin treated animals infected with daptomycin-resistant isolates than in animals infected with the daptomycin-susceptible control isolate, with mean values of 7.5 × 10^5^, 1.3 × 10^6^, 4.5 × 10^7^, and 2.6 × 10^7^ CFU/mL of blood in mice infected with DMG1800332 (MIC = 4 mg/L), DMG1901766 (MIC = 8 mg/L), DMG1700661 (MIC = 16 mg/L), and DMG1700787 (MIC = 16 mg/L), respectively (Figure 4). While the level of VREfm in the blood of daptomycin treated animals infected with DMG1901766 and DMG1800332 was not significantly different, there was significantly higher levels of DMG1700661 and DMG1700787 in the blood of infected mice compared with the control isolate (Figure 4). Based on the mean levels of VREfm in the blood of daptomycin treated mice compared to saline treated control animals there was a 6-fold, 95-fold, and 185-fold reduction in VREfm clearance from the blood of animals infected with DMG1901766, DMG1700787, and DMG1700661, respectively, compared to the daptomycin-susceptible DMG1800332 control isolate. These data indicate that infection with daptomycin-resistant *E. faecium* can compromise the efficacy of daptomycin treatment in the context of bloodstream infections.

## Discussion

Healthcare-associated infections caused by VREfm are a major public health issue globally, with daptomycin being one of the only therapeutic options for treating these infections. Despite this, our understanding of daptomycin resistance in VREfm is incomplete.

A global surveillance study has suggested that the proportion of daptomycin-resistant isolates among VREfm globally may be as low as 0.18% (Sader et al., 2014). However, a meta-analysis of 23 international studies found that the prevalence of daptomycin-resistant VREfm was variable with a range of 0.6–19.5% depending on the study (Kelesidis et al., 2011). In keeping with this, our results suggest that daptomycin-resistant VREfm might be common on the Australasian continent, particularly in Australia where approximately 22% of study isolates were phenotypically resistant to daptomycin. It should be noted that although our study suggests a high rate of daptomycin-resistant VREfm in Australia, the proportion of daptomycin resistance can be influenced by testing method. Further, the non-uniform nature in which isolates were collected means it is not possible to determine an epidemiologically meaningful prevalence rate. As such, future studies should be carried out with isolates that have been collected in an unbiased manner, from multiple sites and over strictly defined timeframes.

Our study identified a previously unreported link between daptomycin resistance and *vanA*-VREfm, with 95% of resistant isolates carrying the *vanA* gene. This finding is of particular concern given the dramatic increase in recent years of *vanA*-VREfm in Australia, where *vanB*-VREfm has traditionally been the most prevalent type (Coombs et al., 2014). Surprisingly, despite the predominance of *vanB*-VREfm in Australia and New Zealand, we did not find any *vanB*-VREfm isolates that displayed a daptomycin-resistant phenotype.

Daptomycin-resistant *vanA*-VREfm were polyclonal, with eight different STs represented. Overall, daptomycin-resistant isolates were phylogenetically diverse, indicating that daptomycin resistance in Australia and New Zealand has not arisen as a consequence of a recent clonal outbreak and has likely emerged independently in multiple VREfm lineages. More in depth sampling and bioinformatics analyses are however needed to confirm this hypothesis. Of note, isolates within the largest clade of daptomycin-resistant study isolates (ST203) were closely related and displayed clear evidence of clonal spread.

It is not clear why *vanA*-VREfm are so strongly associated with daptomycin resistance in our study isolates, especially given the well documented dominance of *vanB*-VREfm in Australia and New Zealand (Lee et al., 2018). It seems unlikely that the *vanA* operon would contribute directly to daptomycin resistance, given the different mechanisms and sites of action of vancomycin and daptomycin. As such, we speculate that different selective pressures may exist for *vanA-*VREfm compared to *vanB*-VREfm, which lead to the selection of daptomycin-resistant *vanA*-VREfm more commonly than *vanB-*VREfm. One possibility is that *vanA*-VREfm are more often exposed to daptomycin, since patients infected with *vanB*-VREfm would likely receive teicoplanin as a first-line therapy in preference to daptomycin.

Previous studies have suggested that *liaFSR* mutations are the dominant mechanism associated with daptomycin resistance in *E. faecium* (Casapao et al., 2013; Diaz et al., 2014). In agreement with this, we identified mutations within *liaSR* in approximately 50% of the daptomycin-resistant study isolates. The co-occurring W73C and T120A mutations in LiaR and LiaS, respectively are arguably the most commonly reported mutations associated with daptomycin-resistance in *E. faecium*, particularly in the United States (Diaz et al., 2014; Davlieva et al., 2018). These mutations were however poorly represented in our isolate collection and were only identified in three isolates. More common amongst our *E. faecium* isolates was the S19F mutation in LiaR, which was identified in 45% of daptomycin-resistant study isolates. Although less common than the W73C LiaR mutation in overseas isolates, the S19F mutation has previously been linked with daptomycin-resistance in an *E. faecium* isolate collected from an immunocompromised patient in Spain (Sorlózano et al., 2015). Our findings suggest that in Australia this mutation may be more dominant than other LiaSR mutations commonly seen overseas. In addition to mutations in the *liaFSR* system, we identified several mutations that were over-represented in our daptomycin-resistant Australian study isolates compared to Australian daptomycin-susceptible study isolates. These were a S63C mutation in DltC, which encodes a D-alanyl carrier protein involved in the D-alanylation of lipoteichoic acid, a S494F mutation in the β-subunit of the RNAP complex and a T641K mutation in the β′-subunit of the RNAP complex. Although these mutations have not previously been linked with daptomycin resistance in *E. faecium*, changes within the *dlt* operon and the RNAP complex have previously been linked with daptomycin-resistance in *S. aureus* (Friedman et al., 2006; Cui et al., 2010; Mishra et al., 2014), providing support for our hypothesis that these mutations might play a role in the emergence of daptomycin resistance in *E. faecium*. The mutations in the β- and β′-subunits of the RNAP complex were most strongly associated with daptomycin resistance, being present in over 60% of daptomycin-resistant study isolates. The co-occurrence of RNAP mutations with the S63C mutation in DltC and the S19F mutation in LiaR in isolates within the ST203 clade raises the possibility of cooperativity between these mutations. Further molecular studies using isogenic mutants are however needed to conclusively determine the role that these individual and combined mutations play in daptomycin resistance in *E. faecium.* Nevertheless, our study adds to a growing body of work suggesting that daptomycin resistance in *E. faecium* is heterogeneous and involves multiple different genes and cellular pathways.

Previous reports have suggested that daptomycin-resistant VREfm can lead to daptomycin treatment failure (Munita et al., 2014), however, there remains a level of uncertainty around the clinical consequences associated with infection by these isolates. To determine whether infection with the predominant daptomycin-resistant clone identified here might impact on daptomycin treatment efficacy, we used a murine VREfm bacteraemia model. These experiments showed that daptomycin treatment was compromised in animals infected with this clone, with the effect being most pronounced in isolates that displayed a daptomycin MIC of 16 mg/L. This is in agreement with some clinical studies that have shown decreased rates of clinical success as the daptomycin MIC increased (Casapao et al., 2013). A recent report documenting the isolation of clinical *vanA*-VREfm isolates possessing daptomycin MICs in the range of 16–32 mg/L are therefore of particular concern (Wang et al., 2018).

The daptomycin dose used in our mouse bacteraemia model has previously been shown to give a level of exposure within the range observed in humans treated with 8 mg/kg/day daptomycin (Heine et al., 2010). This is a commonly used dose in humans infected with VREfm (Casapao et al., 2013; Britt et al., 2017), however, a recent study suggested that high dose daptomycin (≥10 mg/kg/day) might be more effective in treating enterococcal infections (Britt et al., 2017). Further studies on the efficacy of high dose daptomycin for treating infections caused by daptomycin-resistant VREfm are therefore warranted.

## Conclusion

In summary, we have identified a strong association between *vanA*-VREfm and daptomycin resistance. Further, we have identified a number of novel mutations that are over-represented in Australian daptomycin-resistant VREfm compared to daptomycin-susceptible isolates. Finally, using a murine model of VREfm infection, we have shown that infection with the predominant Australian daptomycin-resistant clone significantly impacts on daptomycin treatment efficacy in the context of VREfm bloodstream infections. These findings suggest that clinical decision making regarding the use of daptomycin for infections caused by *vanA*-VREfm should be carefully guided by knowledge of local resistance patterns and supported by phenotypic susceptibility testing of individual *vanA*-VREfm isolates.

## Data Availability Statement

The data presented in the study are deposited in the ENA repository under bioprojects PRJNA433676, PRJNA565795, PRJEB23767, PRJNA565795, and PRJEB47276.

## Ethics Statement

The animal study was reviewed and approved by The University of Melbourne, Department of Biochemistry and Molecular Biology, Dental Science, Medicine, Microbiology & Immunology, and Surgery Animal Ethics Committee.

## Author Contributions

GC and BH conceived and planned the experiments. LL, CH, AT, and YN performed the planned experiments. CG, TS, and TPS provided critical insights into the bioinformatic analyses performed in the study. KD, NS, and DW isolated the VREfm isolates used. LL and GC co-wrote the manuscript with critical feedback and input from all authors.

## Conflict of Interest

The authors declare that the research was conducted in the absence of any commercial or financial relationships that could be construed as a potential conflict of interest.

## Publisher’s Note

All claims expressed in this article are solely those of the authors and do not necessarily represent those of their affiliated organizations, or those of the publisher, the editors and the reviewers. Any product that may be evaluated in this article, or claim that may be made by its manufacturer, is not guaranteed or endorsed by the publisher.

## Footnotes

1
https://github.com/tseemann/shovill

2
https://github.com/tseemann/snippy

3
https://www.R-project.org/

4
https://github.com/tseemann/mlst

5
http://pubmlst.org/efaecium/

6
https://github.com/tseemann/abricate
