# Supplementary material for: Daptomycin Resistance Occurs Predominantly in vanA-Type Vancomycin-Resistant Enterococcus faecium in Australasia and Is Associated With Heterogeneous and Novel Mutations
Source: Front Microbiol. 2021 Oct 20;12:749935. doi: 10.3389/fmicb.2021.749935 (PMC8564391; doi:10.3389/fmicb.2021.749935)
Supplement: Supplementary file 1 [file Table_1.DOCX]

Supplementary Material

# Supplementary Data

Supplementary Table 1: *Enterococcus faecium* study isolates.

| Isolate ID | Daptomycin MIC (mg/L) | van Status | Country | Year | Biosample | Run ID | Bioproject |
| --- | --- | --- | --- | --- | --- | --- | --- |
| AUSMDU00003992 | 8 | VanA | Australia | 2015 | SAMN08595915 | SRR6768174 | PRJNA433676 |
| AUSMDU00003997 | 2 | VanB | Australia | 2015 | SAMN08595920 | SRR6768171 | PRJNA433676 |
| AUSMDU00004003 | 8 | VanA | Australia | 2015 | SAMN08595926 | SRR6768143 | PRJNA433676 |
| AUSMDU00004006 | 2 | VanB | Australia | 2015 | SAMN08595929 | SRR6768146 | PRJNA433676 |
| AUSMDU00004009 | 2 | VanB | Australia | 2015 | SAMN08595932 | SRR6768149 | PRJNA433676 |
| AUSMDU00004022 | 2 | VanB | Australia | 2015 | SAMN08595945 | SRR6768439 | PRJNA433676 |
| AUSMDU00004025 | 2 | VanB | Australia | 2015 | SAMN08595948 | SRR6768237 | PRJNA433676 |
| AUSMDU00004027 | 2 | VanB | Australia | 2015 | SAMN08595950 | SRR6768235 | PRJNA433676 |
| AUSMDU00004060 | 8 | VanA | Australia | 2015 | SAMN08595983 | SRR6768400 | PRJNA433676 |
| AUSMDU00004075 | 4 | VanA | Australia | 2015 | SAMN08595996 | SRR6768470 | PRJNA433676 |
| AUSMDU00004090 | 8 | VanA | Australia | 2015 | SAMN08596011 | SRR6768443 | PRJNA433676 |
| AUSMDU00004107 | 2 | VanB | Australia | 2015 | SAMN08596028 | SRR6768255 | PRJNA433676 |
| AUSMDU00004108 | 1 | VanB | Australia | 2015 | SAMN08596029 | SRR6768256 | PRJNA433676 |
| AUSMDU00004117 | 4 | VanB | Australia | 2015 | SAMN08596038 | SRR6768375 | PRJNA433676 |
| AUSMDU00004124 | 1 | VanB | Australia | 2015 | SAMN08596045 | SRR6768328 | PRJNA433676 |
| AUSMDU00004149 | 8 | VanA | Australia | 2015 | SAMN08596070 | SRR6768427 | PRJNA433676 |
| AUSMDU00004184 | 4 | VanA | Australia | 2015 | SAMN08596105 | SRR6768186 | PRJNA433676 |
| AUSMDU00004212 | 2 | VanB | Australia | 2015 | SAMN08596133 | SRR6768264 | PRJNA433676 |
| AUSMDU00004296 | 8 | VanA | Australia | 2015 | SAMN08596217 | SRR6768231 | PRJNA433676 |
| AUSMDU00004308 | 4 | VanA | Australia | 2015 | SAMN08596229 | SRR6768387 | PRJNA433676 |
| AUSMDU00007435 | 4 | VanA | Australia | 2017 | SAMN12768636 | SRR10126863 | PRJNA565795 |
| AUSMDU00007436 | 4 | VanA | Australia | 2017 | SAMN12768582 | SRR10126859 | PRJNA565795 |
| AUSMDU00007497 | 8 | VanA | Australia | 2017 | SAMN12768583 | SRR10127113 | PRJNA565795 |
| AUSMDU00007498 | 8 | VanA | Australia | 2017 | SAMN12768584 | SRR10126917 | PRJNA565795 |
| AUSMDU00007501 | 8 | VanA | Australia | 2017 | SAMN12768587 | SRR10126820 | PRJNA565795 |
| AUSMDU00007503 | 2 | VanA | Australia | 2017 | SAMN12768637 | SRR10126862 | PRJNA565795 |
| AUSMDU00007995 | 4 | VanA | Australia | 2017 | SAMN12768594 | SRR10127009 | PRJNA565795 |
| AUSMDU00007996 | 4 | VanA | Australia | 2017 | SAMN12768595 | SRR10126970 | PRJNA565795 |
| AUSMDU00007997 | 4 | VanA | Australia | 2017 | SAMN12768596 | SRR10126959 | PRJNA565795 |
| AUSMDU00007998 | 2 | VanA | Australia | 2017 | SAMN12768597 | SRR10126948 | PRJNA565795 |
| AUSMDU00008203 | 1 | VanA | Australia | 2017 | SAMN12768614 | SRR10127068 | PRJNA565795 |
| AUSMDU00008257 | 8 | VanA | Australia | 2017 | SAMN12768641 | SRR10126829 | PRJNA565795 |
| AUSMDU00008378 | 8 | VanA | Australia | 2017 | SAMN12768613 | SRR10127079 | PRJNA565795 |
| AUSMDU00008391 | 4 | VanA | Australia | 2017 | SAMN12768622 | SRR10126878 | PRJNA565795 |
| AUSMDU00008392 | 8 | VanA | Australia | 2017 | SAMN12768644 | SRR10126826 | PRJNA565795 |
| AUSMDU00008393 | 16 | VanA | Australia | 2017 | SAMN12768623 | SRR10126877 | PRJNA565795 |
| AUSMDU00008394 | 8 | VanA | Australia | 2017 | SAMN12768624 | SRR10126876 | PRJNA565795 |
| AUSMDU00008423 | 4 | VanA | Australia | 2017 | SAMN12768628 | SRR10126872 | PRJNA565795 |
| AUSMDU00008569 | 2 | VanA | Australia | 2017 | SAMN12768629 | SRR10126871 | PRJNA565795 |
| AUSMDU00008570 | 8 | VanA | Australia | 2017 | SAMN12768630 | SRR10126869 | PRJNA565795 |
| AUSMDU00008705 | 8 | VanA | Australia | 2015 | SAMEA104415087 | ERR2212581 | PRJEB23767 |
| AUSMDU00008706 | 16 | VanA | Australia | 2015 | SAMEA104415082 | ERR2212576 | PRJEB23767 |
| AUSMDU00008709 | 16 | VanA | Australia | 2015 | SAMEA104415083 | ERR2212577 | PRJEB23767 |
| AUSMDU00008712 | 16 | VanA | Australia | 2015 | SAMEA104415081 | ERR2212575 | PRJEB23767 |
| AUSMDU00010566 | 2 | VanA | Australia | 2017 | SAMN18082637 | SRR13825703 | PRJNA565795 |
| AUSMDU00010570 | 2 | VanA | Australia | 2017 | SAMN18720235 | SRR14673686 | PRJNA565795 |
| AUSMDU00010605 | 8 | VanA | Australia | 2017 | SAMN18082616 | SRR13825599 | PRJNA565795 |
| AUSMDU00010617 | 2 | VanA | Australia | 2017 | SAMN18082690 | SRR13825415 | PRJNA565795 |
| AUSMDU00010640 | 4 | VanA | Australia | 2017 | SAMN18082630 | SRR13825781 | PRJNA565795 |
| AUSMDU00010659 | 2 | VanA | Australia | 2017 | SAMN18082631 | SRR13825770 | PRJNA565795 |
| AUSMDU00010700 | 8 | VanA | Australia | 2017 | SAMN18082676 | SRR13825430 | PRJNA565795 |
| AUSMDU00010701 | 4 | VanA | Australia | 2017 | SAMN18720247 | SRR14673459 | PRJNA565795 |
| AUSMDU00010808 | 8 | VanA | Australia | 2017 | SAMN18082711 | SRR13825392 | PRJNA565795 |
| AUSMDU00010822 | 2 | VanA | Australia | 2017 | SAMN18082686 | SRR13825419 | PRJNA565795 |
| AUSMDU00010910 | 8 | VanA | Australia | 2017 | SAMN18082721 | SRR13825819 | PRJNA565795 |
| AUSMDU00011137 | 8 | VanA | Australia | 2017 | SAMN18082741 | SRR13825797 | PRJNA565795 |
| AUSMDU00011298 | 8 | VanA | Australia | 2017 | SAMN18082775 | SRR13825760 | PRJNA565795 |
| AUSMDU00011340 | 8 | VanA | Australia | 2017 | SAMN18082764 | SRR13825772 | PRJNA565795 |
| AUSMDU00011477 | 4 | VanA | Australia | 2017 | SAMN18082789 | SRR13825744 | PRJNA565795 |
| AUSMDU00011482 | 4 | VanA | Australia | 2017 | SAMN18720242 | SRR14673515 | PRJNA565795 |
| AUSMDU00011555 | 4 | VanA | Australia | 2018 | SAMN18082828 | SRR13825700 | PRJNA565795 |
| AUSMDU00011693 | 4 | VanA | Australia | 2018 | SAMN18082823 | SRR13825706 | PRJNA565795 |
| AUSMDU00011698 | 4 | VanA | Australia | 2018 | SAMN18082810 | SRR13825721 | PRJNA565795 |
| AUSMDU00011707 | 2 | VanA | Australia | 2018 | SAMN18082811 | SRR13825720 | PRJNA565795 |
| AUSMDU00011710 | 4 | VanA | Australia | 2018 | SAMN18082765 | SRR13825771 | PRJNA565795 |
| AUSMDU00011877 | 4 | VanA | Australia | 2018 | SAMN18082853 | SRR13825673 | PRJNA565795 |
| AUSMDU00012084 | 2 | VanA | Australia | 2018 | SAMN18082877 | SRR13825614 | PRJNA565795 |
| AUSMDU00012789 | 4 | VanA | Australia | 2018 | SAMN18082888 | SRR13825602 | PRJNA565795 |
| AUSMDU00014829 | 8 | VanA | Australia | 2018 | SAMN18083108 | SRR13825482 | PRJNA565795 |
| AUSMDU00017846 | 8 | VanA | Australia | 2018 | SAMN18720238 | SRR14673559 | PRJNA565795 |
| AUSMDU00017931 | 8 | VanA | Australia | 2018 | SAMN18085450 | SRR13803231 | PRJNA565795 |
| AUSMDU00018344 | 8 | VanA | Australia | 2018 | SAMN18085497 | SRR13803386 | PRJNA565795 |
| AUSMDU00018395 | 8 | VanA | Australia | 2018 | SAMN18085270 | SRR13802946 | PRJNA565795 |
| AUSMDU00034400 | 2 | VanA | Australia | 2017 | SAMN12768640 | SRR10126830 | PRJNA565795 |
| AUSMDU00034401 | 4 | VanA | Australia | 2017 | SAMN12768601 | SRR10126847 | PRJNA565795 |
| AUSMDU00034402 | 8 | VanA | Australia | 2017 | SAMN12768609 | SRR10127124 | PRJNA565795 |
| AUSMDU00034403 | 2 | VanA | Australia | 2017 | SAMN12768610 | SRR10127112 | PRJNA565795 |
| AUSMDU00034405 | 8 | VanA | Australia | 2017 | SAMN12768598 | SRR10126909 | PRJNA565795 |
| AUSMDU00034406 | 8 | VanA | Australia | 2017 | SAMN12768599 | SRR10126898 | PRJNA565795 |
| AUSMDU00034409 | 8 | VanA | Australia | 2017 | SAMN12768604 | SRR10126786 | PRJNA565795 |
| AUSMDU00034413 | 8 | VanA | Australia | 2017 | SAMN12768612 | SRR10127090 | PRJNA565795 |
| DMG1700755 | 2 | VanB | Australia | 2015 | SAMEA9565987 | ERS7288724 | PRJEB47276 |
| DMG1700756 | 2 | VanB | Australia | 2015 | SAMEA9565988 | ERS7288725 | PRJEB47276 |
| DMG1700757 | 2 | VanB | Australia | 2015 | SAMEA9565989 | ERS7288726 | PRJEB47276 |
| DMG1700758 | 2 | VanB | Australia | 2015 | SAMEA9565990 | ERS7288727 | PRJEB47276 |
| DMG1700759 | 2 | VanB | Australia | 2015 | SAMEA9565991 | ERS7288728 | PRJEB47276 |
| DMG1700760 | 1 | VanB | Australia | 2015 | SAMEA9565975 | ERS7288712 | PRJEB47276 |
| DMG1700761 | 2 | VanB | Australia | 2015 | SAMEA9565992 | ERS7288729 | PRJEB47276 |
| DMG1700762 | 2 | VanB | Australia | 2015 | SAMEA9565993 | ERS7288730 | PRJEB47276 |
| DMG1700763 | 2 | VanB | Australia | 2015 | SAMEA9565994 | ERS7288731 | PRJEB47276 |
| DMG1700764 | 4 | VanB | Australia | 2015 | SAMEA9566036 | ERS7288773 | PRJEB47276 |
| DMG1700765 | 2 | VanB | Australia | 2015 | SAMEA9565995 | ERS7288732 | PRJEB47276 |
| DMG1700766 | 1 | VanB | Australia | 2015 | SAMEA9565976 | ERS7288713 | PRJEB47276 |
| DMG1700767 | 4 | VanB | Australia | 2015 | SAMEA9566037 | ERS7288774 | PRJEB47276 |
| DMG1700768 | 2 | VanB | Australia | 2015 | SAMEA9565996 | ERS7288733 | PRJEB47276 |
| DMG1700769 | 0.5 | VanB | Australia | 2015 | SAMEA9565973 | ERS7288710 | PRJEB47276 |
| DMG1700770 | 2 | VanB | Australia | 2015 | SAMEA9565997 | ERS7288734 | PRJEB47276 |
| DMG1700784 | 4 | VanA | Australia | 2015 | SAMEA9566035 | ERS7288772 | PRJEB47276 |
| DMG1700785 | 8 | VanA | Australia | 2015 | SAMEA9566074 | ERS7288811 | PRJEB47276 |
| DMG1901688 | 4 | VanB | Australia | 2010 | SAMEA9566038 | ERS7288775 | PRJEB47276 |
| DMG1901689 | 1 | VanB | Australia | 2011 | SAMEA9565977 | ERS7288714 | PRJEB47276 |
| DMG1901690 | 1 | VanB | Australia | 2011 | SAMEA9565978 | ERS7288715 | PRJEB47276 |
| DMG1901691 | 2 | VanB | Australia | 2011 | SAMEA9565998 | ERS7288735 | PRJEB47276 |
| DMG1901692 | 2 | VanB | Australia | 2012 | SAMEA9565999 | ERS7288736 | PRJEB47276 |
| DMG1901693 | 4 | VanB | Australia | 2012 | SAMEA9566039 | ERS7288776 | PRJEB47276 |
| DMG1901694 | 2 | VanB | Australia | 2012 | SAMEA9566000 | ERS7288737 | PRJEB47276 |
| DMG1901695 | 0.5 | VanB | Australia | 2012 | SAMEA9565974 | ERS7288711 | PRJEB47276 |
| DMG1901696 | 4 | VanB | Australia | 2013 | SAMEA9566040 | ERS7288777 | PRJEB47276 |
| DMG1901697 | 2 | VanB | Australia | 2014 | SAMEA9566001 | ERS7288738 | PRJEB47276 |
| DMG1901698 | 1 | VanB | Australia | 2014 | SAMEA9565979 | ERS7288716 | PRJEB47276 |
| DMG1901699 | 2 | VanB | Australia | 2014 | SAMEA9566002 | ERS7288739 | PRJEB47276 |
| DMG1901700 | 2 | VanB | Australia | 2014 | SAMEA9566003 | ERS7288740 | PRJEB47276 |
| DMG1901701 | 2 | VanB | Australia | 2014 | SAMEA9566004 | ERS7288741 | PRJEB47276 |
| DMG1901702 | 2 | VanB | Australia | 2014 | SAMEA9566005 | ERS7288742 | PRJEB47276 |
| DMG1901703 | 2 | VanB | Australia | 2015 | SAMEA9566006 | ERS7288743 | PRJEB47276 |
| DMG1901704 | 2 | VanB | Australia | 2015 | SAMEA9566007 | ERS7288744 | PRJEB47276 |
| DMG1901705 | 2 | VanB | Australia | 2015 | SAMEA9566008 | ERS7288745 | PRJEB47276 |
| DMG1901706 | 2 | VanB | Australia | 2015 | SAMEA9566009 | ERS7288746 | PRJEB47276 |
| DMG1901707 | 2 | VanB | Australia | 2015 | SAMEA9566010 | ERS7288747 | PRJEB47276 |
| DMG1901708 | 4 | VanB | Australia | 2015 | SAMEA9566041 | ERS7288778 | PRJEB47276 |
| DMG1901709 | 2 | VanB | Australia | 2015 | SAMEA9566011 | ERS7288748 | PRJEB47276 |
| DMG1901710 | 4 | VanB | Australia | 2015 | SAMEA9566042 | ERS7288779 | PRJEB47276 |
| DMG1901711 | 2 | VanB | Australia | 2015 | SAMEA9566012 | ERS7288749 | PRJEB47276 |
| DMG1901713 | 1 | VanB | Australia | 2015 | SAMEA9565980 | ERS7288717 | PRJEB47276 |
| DMG1901714 | 1 | VanB | Australia | 2015 | SAMEA9565981 | ERS7288718 | PRJEB47276 |
| DMG1901715 | 1 | VanB | Australia | 2015 | SAMEA9565982 | ERS7288719 | PRJEB47276 |
| DMG1901716 | 1 | VanB | Australia | 2015 | SAMEA9565983 | ERS7288720 | PRJEB47276 |
| DMG1901717 | 1 | VanB | Australia | 2015 | SAMEA9565984 | ERS7288721 | PRJEB47276 |
| DMG1901718 | 1 | VanB | Australia | 2015 | SAMEA9565985 | ERS7288722 | PRJEB47276 |
| DMG1901719 | 2 | VanB | Australia | 2011 | SAMEA9566013 | ERS7288750 | PRJEB47276 |
| DMG1901720 | 2 | VanB | Australia | 2011 | SAMEA9566014 | ERS7288751 | PRJEB47276 |
| DMG1901721 | 2 | VanB | Australia | 2011 | SAMEA9566015 | ERS7288752 | PRJEB47276 |
| DMG1901722 | 2 | VanB | Australia | 2012 | SAMEA9566016 | ERS7288753 | PRJEB47276 |
| DMG1901723 | 2 | VanB | Australia | 2013 | SAMEA9566017 | ERS7288754 | PRJEB47276 |
| DMG1901724 | 2 | VanB | Australia | 2014 | SAMEA9566018 | ERS7288755 | PRJEB47276 |
| DMG1901725 | 2 | VanB | Australia | 2014 | SAMEA9566019 | ERS7288756 | PRJEB47276 |
| DMG1901726 | 1 | VanB | Australia | 2014 | SAMEA9565986 | ERS7288723 | PRJEB47276 |
| DMG1901727 | 8 | VSE | Australia | 2007 | SAMEA9565971 | ERS7288708 | PRJEB47276 |
| DMG1901728 | 2 | VSE | Australia | 2007 | SAMEA9566020 | ERS7288757 | PRJEB47276 |
| DMG1901729 | 4 | VSE | Australia | 2007 | SAMEA9566043 | ERS7288780 | PRJEB47276 |
| DMG1901730 | 4 | VSE | Australia | 2007 | SAMEA9566044 | ERS7288781 | PRJEB47276 |
| DMG1901731 | 2 | VSE | Australia | 2008 | SAMEA9566021 | ERS7288758 | PRJEB47276 |
| DMG1901732 | 2 | VSE | Australia | 2008 | SAMEA9566022 | ERS7288759 | PRJEB47276 |
| DMG1901733 | 4 | VSE | Australia | 2009 | SAMEA9566045 | ERS7288782 | PRJEB47276 |
| DMG1901734 | 4 | VSE | Australia | 2009 | SAMEA9566046 | ERS7288783 | PRJEB47276 |
| DMG1901735 | 4 | VSE | Australia | 2009 | SAMEA9566047 | ERS7288784 | PRJEB47276 |
| DMG1901736 | 4 | VSE | Australia | 2009 | SAMEA9566048 | ERS7288785 | PRJEB47276 |
| DMG1901737 | 4 | VSE | Australia | 2009 | SAMEA9566049 | ERS7288786 | PRJEB47276 |
| DMG1901738 | 4 | VSE | Australia | 2009 | SAMEA9566050 | ERS7288787 | PRJEB47276 |
| DMG1901739 | 2 | VSE | Australia | 2010 | SAMEA9566023 | ERS7288760 | PRJEB47276 |
| DMG1901740 | 2 | VSE | Australia | 2010 | SAMEA9566024 | ERS7288761 | PRJEB47276 |
| DMG1901741 | 4 | VSE | Australia | 2010 | SAMEA9566051 | ERS7288788 | PRJEB47276 |
| DMG1901742 | 2 | VSE | Australia | 2011 | SAMEA9566025 | ERS7288762 | PRJEB47276 |
| DMG1901743 | 4 | VSE | Australia | 2011 | SAMEA9566052 | ERS7288789 | PRJEB47276 |
| DMG1901744 | 4 | VSE | Australia | 2011 | SAMEA9566053 | ERS7288790 | PRJEB47276 |
| DMG1901745 | 4 | VSE | Australia | 2011 | SAMEA9566054 | ERS7288791 | PRJEB47276 |
| DMG1901746 | 4 | VSE | Australia | 2011 | SAMEA9566055 | ERS7288792 | PRJEB47276 |
| DMG1901747 | 8 | VSE | Australia | 2011 | SAMEA9565972 | ERS7288709 | PRJEB47276 |
| DMG1901748 | 2 | VSE | Australia | 2012 | SAMEA9566026 | ERS7288763 | PRJEB47276 |
| DMG1901749 | 2 | VSE | Australia | 2012 | SAMEA9566027 | ERS7288764 | PRJEB47276 |
| DMG1901750 | 2 | VSE | Australia | 2013 | SAMEA9566028 | ERS7288765 | PRJEB47276 |
| DMG1901751 | 4 | VSE | Australia | 2013 | SAMEA9566056 | ERS7288793 | PRJEB47276 |
| DMG1901752 | 4 | VSE | Australia | 2015 | SAMEA9566057 | ERS7288794 | PRJEB47276 |
| DMG1901753 | 2 | VSE | Australia | 2015 | SAMEA9566029 | ERS7288766 | PRJEB47276 |
| DMG1901754 | 2 | VSE | Australia | 2015 | SAMEA9566030 | ERS7288767 | PRJEB47276 |
| DMG1901755 | 4 | VSE | Australia | 2015 | SAMEA9566058 | ERS7288795 | PRJEB47276 |
| DMG1901756 | 4 | VSE | Australia | 2008 | SAMEA9566059 | ERS7288796 | PRJEB47276 |
| DMG1901757 | 4 | VSE | Australia | 2008 | SAMEA9566060 | ERS7288797 | PRJEB47276 |
| DMG1901758 | 2 | VSE | Australia | 2008 | SAMEA9566031 | ERS7288768 | PRJEB47276 |
| DMG1901759 | 2 | VSE | Australia | 2008 | SAMEA9566032 | ERS7288769 | PRJEB47276 |
| DMG1901760 | 2 | VSE | Australia | 2009 | SAMEA9566033 | ERS7288770 | PRJEB47276 |
| DMG1901761 | 2 | VSE | Australia | 2013 | SAMEA9566034 | ERS7288771 | PRJEB47276 |
| DMG1901762 | 4 | VSE | Australia | 2013 | SAMEA9566061 | ERS7288798 | PRJEB47276 |
| DMG1901763 | 4 | VSE | Australia | 2014 | SAMEA9566062 | ERS7288799 | PRJEB47276 |
| DMG1901764 | 4 | VSE | Australia | 2008 | SAMEA9566063 | ERS7288800 | PRJEB47276 |
| DMG1700792 | 4 | VanB | New Zealand | 2014 |  |  |  |
| DMG1700793 | 4 | VanB | New Zealand | 2014 |  |  |  |
| DMG1700794 | 4 | VanB | New Zealand | 2014 |  |  |  |
| DMG1901836 | 4 | VanA | New Zealand | 2014 |  |  |  |
| DMG1901837 | 4 | VanA | New Zealand | 2014 |  |  |  |
| DMG1901838 | 4 | VanA | New Zealand | 2014 |  |  |  |
| DMG1901839 | 4 | VanA | New Zealand | 2014 |  |  |  |
| DMG1901840 | 4 | VanA | New Zealand | 2014 |  |  |  |
| DMG1901841 | 4 | VanA | New Zealand | 2014 |  |  |  |
| DMG1901842 | 4 | VanA | New Zealand | 2014 |  |  |  |
| DMG1901845 | 4 | VanA | New Zealand | 2014 |  |  |  |
| DMG1901846 | 4 | VanA | New Zealand | 2014 |  |  |  |
| DMG1901848 | 4 | VanA | New Zealand | 2014 |  |  |  |
| DMG1901849 | 4 | VanA | New Zealand | 2013 |  |  |  |
| DMG1901850 | 4 | VanA | New Zealand | 2013 |  |  |  |
| DMG1901851 | 4 | VanA | New Zealand | 2013 |  |  |  |
| DMG1901852 | 4 | VanA | New Zealand | 2013 |  |  |  |
| DMG1901853 | 4 | VanA | New Zealand | 2013 |  |  |  |
| DMG1901854 | 4 | VanA | New Zealand | 2013 |  |  |  |
| DMG1901856 | 4 | VanA | New Zealand | 2013 |  |  |  |
| DMG1901857 | 4 | VanA | New Zealand | 2013 |  |  |  |
| DMG1901858 | 2 | VanA | New Zealand | 2013 |  |  |  |
| DMG1901859 | 4 | VanA | New Zealand | 2013 |  |  |  |
| DMG1901860 | 4 | VanA | New Zealand | 2013 |  |  |  |
| DMG1901861 | 4 | VanA | New Zealand | 2013 |  |  |  |
| DMG1901862 | 4 | VanA | New Zealand | 2013 |  |  |  |
| DMG1901863 | 4 | VanA | New Zealand | 2013 |  |  |  |
| DMG1901864 | 4 | VanA | New Zealand | 2013 |  |  |  |
| DMG1901865 | 4 | VanA | New Zealand | 2013 |  |  |  |
| DMG1901866 | 4 | VanA | New Zealand | 2013 |  |  |  |
| DMG1901867 | 4 | VanA | New Zealand | 2013 |  |  |  |
| DMG1901868 | 2 | VanA | New Zealand | 2013 |  |  |  |
| DMG1901869 | 4 | VanA | New Zealand | 2013 |  |  |  |
| DMG1901870 | 4 | VanA | New Zealand | 2013 |  |  |  |
| DMG1901872 | 4 | VanA | New Zealand | 2013 |  |  |  |
| DMG1901873 | 4 | VanA | New Zealand | 2013 |  |  |  |
| DMG1901874 | 4 | VanA | New Zealand | 2013 |  |  |  |
| DMG1901875 | 4 | VanA | New Zealand | 2013 |  |  |  |
| DMG1901876 | 4 | VanA | New Zealand | 2013 |  |  |  |
| DMG1901877 | 4 | VanA | New Zealand | 2013 |  |  |  |
| DMG1901878 | 4 | VanA | New Zealand | 2013 |  |  |  |
| DMG1901879 | 4 | VanA | New Zealand | 2013 |  |  |  |
| DMG1901880 | 4 | VanA | New Zealand | 2013 |  |  |  |
| DMG1901881 | 4 | VanA | New Zealand | 2013 |  |  |  |
| DMG1901882 | 4 | VanA | New Zealand | 2013 |  |  |  |
| DMG1901883 | 4 | VanA | New Zealand | 2013 |  |  |  |
| DMG1901884 | 4 | VanA | New Zealand | 2013 |  |  |  |
| DMG1901885 | 4 | VanA | New Zealand | 2013 |  |  |  |
| DMG1901886 | 4 | VanA | New Zealand | 2013 |  |  |  |
| DMG1901887 | 4 | VanA | New Zealand | 2013 |  |  |  |
| DMG1901889 | 4 | VanB | New Zealand | 2014 |  |  |  |
| DMG1901890 | 4 | VanB | New Zealand | 2014 |  |  |  |
| DMG1901891 | 4 | VanB | New Zealand | 2014 |  |  |  |
| DMG1901892 | 4 | VanB | New Zealand | 2014 |  |  |  |
| DMG1901893 | 4 | VanB | New Zealand | 2014 |  |  |  |
| DMG1901894 | 4 | VanB | New Zealand | 2014 |  |  |  |
| DMG1901895 | 4 | VanB | New Zealand | 2014 |  |  |  |
| DMG1901896 | 4 | VanB | New Zealand | 2014 |  |  |  |
| DMG1901897 | 4 | VanB | New Zealand | 2014 |  |  |  |
| DMG1901898 | 4 | VanB | New Zealand | 2014 |  |  |  |
| DMG1901899 | 4 | VanB | New Zealand | 2014 |  |  |  |
| DMG1901900 | 4 | VanB | New Zealand | 2014 |  |  |  |
| DMG1901901 | 4 | VanB | New Zealand | 2014 |  |  |  |
| DMG1901902 | 4 | VanB | New Zealand | 2014 |  |  |  |
| DMG1901903 | 4 | VanB | New Zealand | 2014 |  |  |  |
| DMG1901904 | 4 | VanB | New Zealand | 2014 |  |  |  |
| DMG1901905 | 4 | VanB | New Zealand | 2014 |  |  |  |
| DMG1901906 | 4 | VanB | New Zealand | 2014 |  |  |  |
| DMG1901907 | 4 | VanB | New Zealand | 2013 |  |  |  |
| DMG1901908 | 2 | VanB | New Zealand | 2013 |  |  |  |
| DMG1901909 | 2 | VanB | New Zealand | 2013 |  |  |  |
| DMG1901910 | 1 | VanB | New Zealand | 2013 |  |  |  |
| DMG1901911 | 4 | VanB | New Zealand | 2013 |  |  |  |
| DMG1901912 | 4 | VanB | New Zealand | 2013 |  |  |  |
| DMG1901913 | 1 | VanB | New Zealand | 2013 |  |  |  |
| DMG1901914 | 1 | VanB | New Zealand | 2013 |  |  |  |
| DMG1901915 | 1 | VanB | New Zealand | 2013 |  |  |  |
| DMG1901916 | 1 | VanB | New Zealand | 2013 |  |  |  |
| DMG1901917 | 4 | VanB | New Zealand | 2013 |  |  |  |
| DMG1901918 | 4 | VanB | New Zealand | 2013 |  |  |  |
| DMG1901919 | 4 | VanB | New Zealand | 2013 |  |  |  |
| DMG1901920 | 4 | VanB | New Zealand | 2013 |  |  |  |
| DMG1901921 | 2 | VanB | New Zealand | 2013 |  |  |  |
| DMG1901922 | 1 | VanB | New Zealand | 2013 |  |  |  |
| DMG1901923 | 4 | VanB | New Zealand | 2013 |  |  |  |
| DMG1901924 | 2 | VanB | New Zealand | 2013 |  |  |  |
| DMG1901925 | 2 | VanB | New Zealand | 2013 |  |  |  |
| DMG1901926 | 4 | VanB | New Zealand | 2013 |  |  |  |
| DMG1901927 | 1 | VanB | New Zealand | 2013 |  |  |  |
| DMG1901928 | 4 | VanB | New Zealand | 2013 |  |  |  |
| DMG1901929 | 2 | VanB | New Zealand | 2013 |  |  |  |
| DMG1901930 | 4 | VanB | New Zealand | 2013 |  |  |  |
| DMG1901931 | 4 | VanB | New Zealand | 2013 |  |  |  |
| DMG1901932 | 4 | VanB | New Zealand | 2013 |  |  |  |
| DMG1901933 | 1 | VanB | New Zealand | 2013 |  |  |  |
| DMG1901934 | 1 | VanB | New Zealand | 2013 |  |  |  |
| DMG1901935 | 1 | VanB | New Zealand | 2013 |  |  |  |
| DMG1901936 | 2 | VanB | New Zealand | 2013 |  |  |  |
| DMG1901937 | 2 | VanB | New Zealand | 2013 |  |  |  |
| DMG1901938 | 1 | VanB | New Zealand | 2013 |  |  |  |
| DMG1901939 | 2 | VanB | New Zealand | 2013 |  |  |  |
| DMG1901940 | 4 | VanB | New Zealand | 2013 |  |  |  |
| DMG1901941 | 4 | VanB | New Zealand | 2013 |  |  |  |
| DMG1901942 | 2 | VanB | New Zealand | 2013 |  |  |  |
| DMG1901943 | 4 | VanB | New Zealand | 2013 |  |  |  |
| DMG1901944 | 4 | VanB | New Zealand | 2013 |  |  |  |
| DMG1901945 | 4 | VanB | New Zealand | 2013 |  |  |  |
| DMG1901946 | 2 | VanB | New Zealand | 2013 |  |  |  |
| DMG1901947 | 4 | VanB | New Zealand | 2013 |  |  |  |
| DMG1901948 | 4 | VanB | New Zealand | 2013 |  |  |  |
| DMG1901949 | 2 | VanB | New Zealand | 2013 |  |  |  |
| DMG1901950 | 4 | VanB | New Zealand | 2013 |  |  |  |
| DMG1901951 | 2 | VanB | New Zealand | 2013 |  |  |  |
| DMG1901952 | 2 | VanB | New Zealand | 2013 |  |  |  |
| DMG1901953 | 4 | VanB | New Zealand | 2013 |  |  |  |
| DMG1901954 | 1 | VanB | New Zealand | 2013 |  |  |  |
| DMG1901955 | 4 | VanB | New Zealand | 2013 |  |  |  |
| DMG1901956 | 4 | VanB | New Zealand | 2013 |  |  |  |
| DMG1901957 | 2 | VanB | New Zealand | 2013 |  |  |  |
| DMG1901958 | 4 | VanB | New Zealand | 2013 |  |  |  |
| DMG1901959 | 4 | VanB | New Zealand | 2013 |  |  |  |
| DMG1901960 | 4 | VanB | New Zealand | 2013 |  |  |  |
| DMG1901961 | 4 | VanB | New Zealand | 2013 |  |  |  |
| DMG1901962 | 2 | VanB | New Zealand | 2013 |  |  |  |
| DMG1901963 | 4 | VanB | New Zealand | 2013 |  |  |  |
| DMG1901964 | 4 | VanB | New Zealand | 2013 |  |  |  |
| DMG1901965 | 2 | VanB | New Zealand | 2013 |  |  |  |
| DMG1901966 | 4 | VanB | New Zealand | 2013 |  |  |  |
| DMG1901967 | 4 | VanB | New Zealand | 2013 |  |  |  |
| DMG1901968 | 4 | VanB | New Zealand | 2013 |  |  |  |
| DMG1901969 | 4 | VanB | New Zealand | 2013 |  |  |  |
| DMG1901970 | 4 | VanB | New Zealand | 2013 |  |  |  |
| DMG1901971 | 4 | VanB | New Zealand | 2013 |  |  |  |
| DMG1901972 | 2 | VanB | New Zealand | 2013 |  |  |  |
| DMG1901973 | 2 | VanB | New Zealand | 2013 |  |  |  |
| DMG1901974 | 2 | VanB | New Zealand | 2013 |  |  |  |
| DMG1901975 | 4 | VanB | New Zealand | 2013 |  |  |  |
| DMG1901976 | 2 | VanB | New Zealand | 2013 |  |  |  |
| DMG1901977 | 4 | VanB | New Zealand | 2013 |  |  |  |
| DMG1901978 | 4 | VanB | New Zealand | 2014 |  |  |  |
| DMG1903253 | 8 | VanA | New Zealand | 2014 | SAMEA9566064 | ERS7288801 | PRJEB47276 |
| DMG1903254 | 8 | VanA | New Zealand | 2014 | SAMEA9566065 | ERS7288802 | PRJEB47276 |
| DMG1903255 | 8 | VanA | New Zealand | 2014 | SAMEA9566066 | ERS7288803 | PRJEB47276 |
| DMG1903256 | 8 | VanA | New Zealand | 2014 | SAMEA9566067 | ERS7288804 | PRJEB47276 |
| DMG1903257 | 8 | VanA | New Zealand | 2014 | SAMEA9566068 | ERS7288805 | PRJEB47276 |
| DMG1903258 | 8 | VanA | New Zealand | 2014 | SAMEA9566069 | ERS7288806 | PRJEB47276 |
| DMG1903259 | 8 | VanA | New Zealand | 2014 | SAMEA9566070 | ERS7288807 | PRJEB47276 |
| DMG1903260 | 8 | VanA | New Zealand | 2013 | SAMEA9566071 | ERS7288808 | PRJEB47276 |
| DMG1903261 | 8 | VanA | New Zealand | 2013 | SAMEA9566072 | ERS7288809 | PRJEB47276 |
| DMG1903262 | 8 | VanA | New Zealand | 2014 | SAMEA9566073 | ERS7288810 | PRJEB47276 |

Supplementary Table 2: Proteins and corresponding mutations associated with daptomycin-resistance that study isolates were computational screened for.

| Protein | Daptomycin resistance associated mutations |
| --- | --- |
| LiaR | S19F, W73C |
| LiaS | L70F, T120A, D251N |
| LiaF | T35I, L39F, I142T, N110S |
| ClsA | N13I, N13T, A20D, D27N, T38C, R54H, R211L, H215R, R218Q, R267H, T644C |
| MprF | T416P, K744E, A760S |
| YycG | S333L, A414T |
| YycH | D311E, G386R, N429S |
| YycI | N185S, G228Q |
| DivIVA | Q73A |
| OatA | E480STOP |
